# Effect of AgNPs on PLA-Based Biocomposites with Polysaccharides: Biodegradability, Antibacterial Activity and Features

**DOI:** 10.3390/ijms262210916

**Published:** 2025-11-11

**Authors:** Kristine V. Aleksanyan, Elena E. Mastalygina, Regina S. Smykovskaya, Nadezhda A. Samoilova, Viktor A. Novikov, Aleksander M. Shakhov, Yana V. Ryzhmanova, Galina A. Kochkina, Natalya E. Ivanushkina

**Affiliations:** 1 Plekhanov Russian University of Economics, 36 Stremyanny Ln, Moscow 117997, Russia; elena.mastalygina@gmail.com; 2 Semenov Federal Research Center for Chemical Physics, Russian Academy of Sciences, 4 Kosygina St., Moscow 119991, Russia; 3 Emanuel Institute of Biochemical Physics, Russian Academy of Sciences, 4 Kosygina St., Moscow 119991, Russia; 4 Nesmeyanov Institute of Organoelement Compounds, Russian Academy of Sciences, 28 Bld. 1 Vavilova St., Moscow 119334, Russia; 5 Skryabin Institute of Biochemistry and Physiology of Microorganisms, Federal Research Center “Pushchino Scientific Center for Biological Research of the Russian Academy of Sciences”, pr. Nauki 5, Pushchino 142292, Moscow Region, Russiagaga56@mail.ru (G.A.K.); ivanushkina58@mail.ru (N.E.I.)

**Keywords:** poly(lactic acid) PLA, polysaccharides, silver nanoparticles, biodegradability, antibacterial activity

## Abstract

According to existing ecological problems, one of the promising developments is the creation of polyfunctional materials, which can be biodegradable, along with possessing antibacterial activity. The present research proposes biocomposites based on PLA with silver nanoparticles (AgNPs) and natural polysaccharides obtained in a twin-screw extruder. Introduction of polysaccharides to PLA-based biocomposites with/without AgNPs led to significant decrease in the elastic modulus and tensile strength, while the elongation at break remained almost unchanged. Thanks to the presence of natural polysaccharides, there was intensified biodegradation in soil despite the AgNP availability. The maximal mass loss was 29% for the PLA–PEG_1000_–starch + AgNPs (80:10:10 + 0.5 wt%) biocomposite. Analyses of the systems before and after soil exposure were carried out using DSC and FTIR spectroscopy methods. According to a thermal analysis, it was found that PLA crystalline regions degrade during exposure to soil. The same feature was detected during the spectral analysis. The intensity of the characteristic absorption bands of PLA decreased. Furthermore, it was found that the dark areas on the surface of the materials are of a polysaccharide nature and may be signs of biofouling of the materials by microbial flora. The tests on fungus resistance showed that biocidal additives such as AgNPs in PLA-based biocomposites with polysaccharides did not inhibit the development of mycelial fungi–biodestructors. And the increased amount of chitosan in the films contributed to their more active destruction by the end of the observation period. It was demonstrated that such biocomposites can inhibit bacterial growth.

## 1. Introduction

Currently, there is a high demand for new polymeric materials with antimicrobial activity that can suppress the growth and reproduction of microorganisms [1]. For these purposes, one of the most attractive polymers is poly(lactic acid) (PLA), which can be widely used in the production of food packaging and biomedical materials and for the development of other effective antimicrobial systems. At the same time, the trend toward research into the creation of biodegradable systems does not lose its relevance, and is increasingly enshrined in law in various countries, for example, in the Environmental Protection Agency (EPA) in the United States [2]. There are a lot of directives in Europe to control the production of eco-friendly materials, for example, the Single Use Plastics Directive, the Plastic Carrier Bags Directive, the Packaging and Packaging Waste Directive and the Waste Framework Directive, which address some aspects and applications of biobased, biodegradable and compostable plastics [3,4,5]. The PLA degradation is a complex mechanism determined by the properties of the polymer, the ambient temperature and the set of microorganisms in the environment. For example, PLA degradation in a natural aquatic environment can take 12 to 16 months [6]. Therefore, the introduction of various additives to the raw materials for the production of plastics, which, on the one hand, can increase the biocidal properties of plastic, and, on the other hand, will contribute to the acceleration of the process of its degradation in nature, is extremely important.

In recent years, various substances have been incorporated into the PLA polymer matrix, including natural compounds, peptides, enzymes, chelating agents and antibiotics. Essential oils and nanoparticles of various metals and their oxides—titanium, zinc, silver—also act as antimicrobial additives [7,8]. So, authors [9] developed PLA-based systems containing Cu^2+^(Fe^3+^) complexes for the creation of medical-purpose materials, which have complete biocidal activity against S. aureus, with complete suppression of bacterial growth occurring within 3 h. The development of biomaterials for regenerative medicine purposes is also associated with the use of nanoparticles of various metals in PLA nanofibers [10]. According to the obtained research data on their vapor permeability, biodegradability and conductivity, these hybrid materials containing ZnO, Fe_3_O_4_ and Au meet the requirements for skin tissue matrices that promote wound healing, along with the absence of cytotoxicity.

The antimicrobial properties of silver are well known, so silver nanoparticles are increasingly being incorporated into polymer matrices, in particular PLA, to provide biocidal properties [11,12,13,14,15,16,17,18]. Nanocomposite films with PLA and various amounts of silver (up to 0.5 wt%) demonstrated high antimicrobial activity against *Escherichia coli* and *Listeria monocytogenes* bacteria [19]. In turn, high efficiency against *S. aureus* and *E. coli* was demonstrated in studies on packaging materials based on PLA with the addition of 1% wt/wt AgNPs in tests with strawberries [20]. Recently developed biodegradable hybrid materials based on PLA with the addition of carbon black and silver nanoparticles have not only improved their mechanical properties and electrical conductivity but also achieved a high antimicrobial effect against a wide range of prokaryotes and eukaryotes—*Bacillus subtilis*, *Micrococcus luteus*, *E. coli*, *Pseudomonas aeruginosa* and *Candida albicans*—which made it possible to use them in a new application as materials for computer keyboards [13].

Currently, a large number of scientific studies are devoted to bridging the gap between innovations in this area and the practical application of research results in the development of new generation materials [21]. Not the least of the problems is the issue of the downside of the biocidal properties of silver nanoparticles and, as a consequence, the possible reduction in the biodegradability of materials with their inclusion. The main biodegraders of polymeric materials in nature are mycelial fungi, so the task of assessing their destruction of materials with the inclusion of silver nanoparticles is relevant. At the same time, the effect of silver nanoparticles on fungi has not been sufficiently studied. On the one hand, studies conducted on yeast show that silver ions can cause significant changes in the cell membrane, including changes in the permeability and destruction of lipid bilayers, as well as suppression of the sporulation process, a decrease in the production of mycotoxins and increased vacuolization [22,23]. On the other hand, in the presence of information on the inhibition of the activity of individual enzymes, there is data on the increase in the general enzymatic activity of mold fungi under the action of silver ions [23]. A study of the effect of silver nanoparticles on the metabolic activity of fungi showed that silver ions can affect fungi selectively. In particular, they activated the formation of extracellular polyphenoloxidases in the fungus *Phanerochaete chrysosporium* and simultaneously suppressed the growth of other fungi and bacteria [24].

An important factor in the creation of biodegradable materials is their ability to decompose in natural conditions. For several decades, a large number of studies have been devoted to the creation of such materials, in which the main role of the initiator of biodegradation is played by natural polysaccharides (for example, [17,18,25,26,27,28,29,30,31,32]). It should be noted that the most popular polysaccharide in this area is starch (which is obtained from various sources (potato, rice, cassava)), which is due to a number of factors: availability, renewability and cheapness. The number of works with starch is huge. In particular, the authors also previously worked on PLA systems with starch [33,34]. Meanwhile the research with chitosan is more often aimed at applications in the medical field as drug carriers and coating products, as well as for the creation of various implants, including those obtained using additive technologies [29]. In addition, systems containing chitosan and PLA with the addition of silver nanowires (at an optimal content of 3 wt%) can be successfully used as cartridges for water filtration, including those with the ability to suppress pathogenic microflora [35].

In this regard, the aim of this work was to create biocomposites based on plasticized PLA with an antimicrobial agent (AgNPs) and cheap and accessible polysaccharides (starch and chitosan), promoting the acceleration of biodegradation, which could meet the modern challenges of polymer chemistry regarding obtaining multifunctional materials used in various fields.

## 2. Results and Discussion

### 2.1. Production of Biocomposites and Their Mechanical and Structural Features

The technique and features of AgNP synthesis was described in [36]. As a result, golden-brown powder of AgNPs stabilized by poly(ethylene-alt-maleic acid (anhydride)) was obtained with a size of about 40–60 nm (Figure 1 in [36]). The PLA-based biocomposites with and without AgNPs with different polysaccharides (starch, chitosan) were obtained in a twin-screw microextruder under the joint action of shear deformations and high temperature. First, PLA granules were loaded into an extruder to obtain the polymer melt, then PEG with polysaccharide or PEG mixed with AgNPs and polysaccharide were introduced into the PLA melt. The PLA content was varied from 70 to 80 wt%, PEG 10 wt%, polysaccharide (starch, chitosan) from 10 to 20 wt% and AgNPs from 0.1 to 0.5 wt% to form the PLA–PEG–polysaccharide biocomposite weight. In order to explore the properties of the final biocomposites, the films were pressed.

The mechanical characteristics were investigated in stretching mode. Analysis of the mechanical characteristics of the PLA-based biocomposites showed that the introduction of polysaccharides led to a significant fall in the elastic modulus *E* and ultimate tensile strength *σ_b_*, while the elongation at break *ε_b_* changed slightly (Table 1). Pure PLA treated in a twin-screw microextruder was also tested. Regarding the example of biocompostes with starch, we found that the presence of AgNPs drastically affected the mechanical characteristics. So, the introduction and rise in content of AgNPs resulted in a rise in *E*, while *σ_b_* first fell and then increased at the maximal content of nanoparticles. At the same time, the changes in elongation at break were not pronounced. Similar regularities in these characteristics were also detected for biocomposites with chitosan at different content of AgNPs. Such an effect can be dictated by both the nature of polysaccharides (rigid polymers) and content of AgNPs.

**Figure 1:**
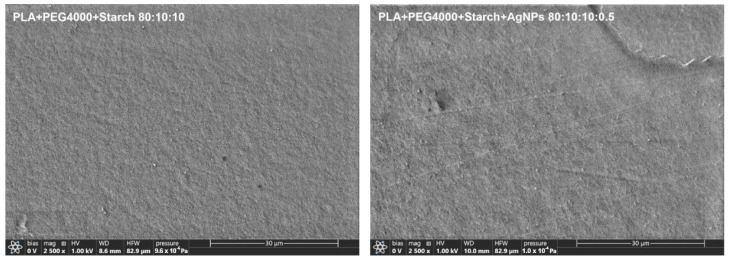
Micrographs of surface for PLA–PEG_4000_–starch (80:10:10 wt%) and PLA–PEG_4000_–starch + AgNPs (80:10:10 + 0.5 wt%).

The morphologies of the samples were studied using the SEM method, where the samples were photographed in raster mode. The surface inspection of biocomposites based on PLA with PEG and starch showed the absence of the effect of the addition of AgNPs at a concentration of 0.5 wt% (Figure 1).

**Figure 2:**
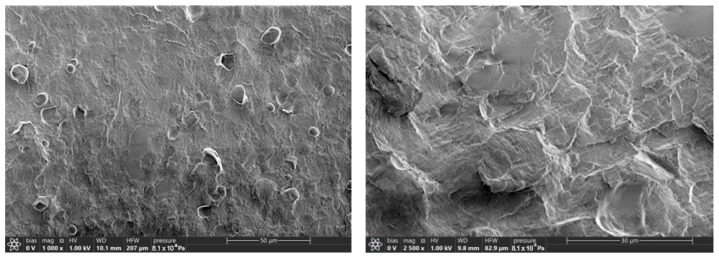
Micrographs of cross-sections for PLA–PEG_1000_–starch (80:10:10 wt%) at different magnifications.

The cross-sections of the samples were obtained in liquid nitrogen. There were no significant differences found when comparing the cross-section morphology of the samples with PEG of different molecular weights (as an example, PLA–PEG_1000_–starch (80:10:10 wt%) cross-sections are presented in Figure 2). Starch granules are visible; they are uniformly distributed in the matrix of plasticized PLA.

**Figure 3:**
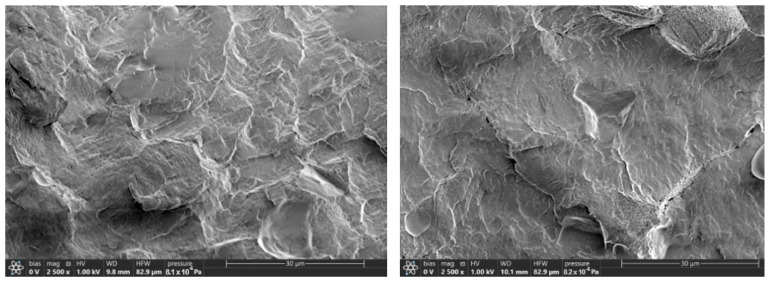
Micrographs of cross-section of PLA–PEG_4000_–starch + AgNPs (80:10:10 + 0.5 wt%) at different magnifications.

Figure 3 shows micrographs of PLA–PEG_4000_–starch + AgNPs (80:10:10 + 0.5 wt%) cross-sections. The images show also that starch is uniformly distributed in the PLA matrix plasticized by PEG, but AgNPs are not visualized at their maximal content. Thus, it can be assumed that AgNPs in the biocomposite containing PEG, which acts not only as a plasticizer for PLA but also as an additional stabilizer for nanoparticles, as well as a polysaccharide, are encapsulated in the components of the system.

### 2.2. Tests on Exposure in Soil

**Figure 4:**
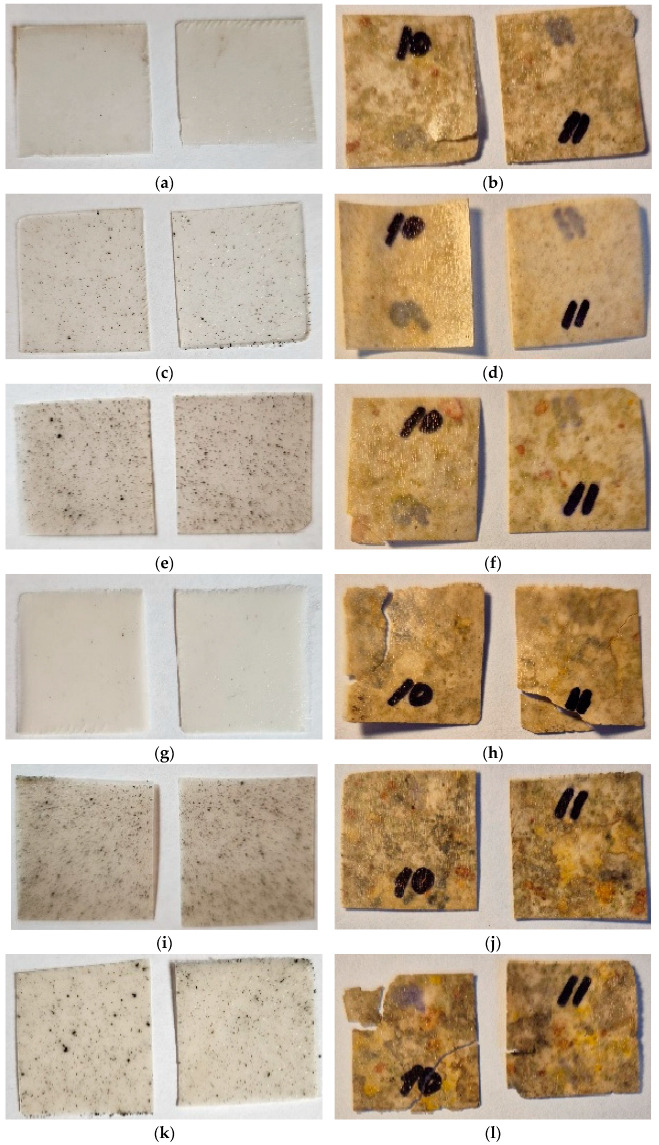
Photographs of films before (**a**,**c**,**e**,**g**,**i**,**k**) and after exposure to soil for 6 months (**b**,**d**,**f**,**h**,**j**,**l**): PLA–PEG_1000_–starch (80:10:10 wt%) (**a**,**b**); PLA–PEG_1000_–starch + AgNPs (80:10:10 + 0.1 wt%) (**c**,**d**); PLA–PEG_1000_–starch + AgNPs (80:10:10 + 0.5 wt%) (**e**,**f**); PLA–PEG_1000_–starch (70:10:20 wt%) (**g**,**h**); PLA–PEG_1000_–starch + AgNPs (70:10:20 + 0.5 wt%) (**i**,**j**); PLA–PEG_4000_–starch + AgNPs (70:10:20 + 0.5 wt%) (**k**,**l**).

**Figure 5:**
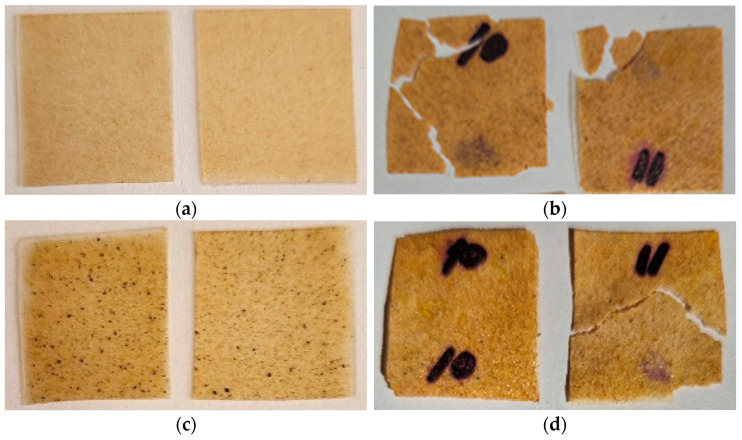
Photographs of films before (**a**,**c**) and after exposure to soil for 8 months (**b**,**d**): PLA–PEG_1000_–chitosan (80:10:10 wt%) (**a**,**b**); PLA–PEG_1000_–chitosan + AgNPs (80:10:10 + 0.5 wt%) (**c**,**d**).

**Figure 6:**
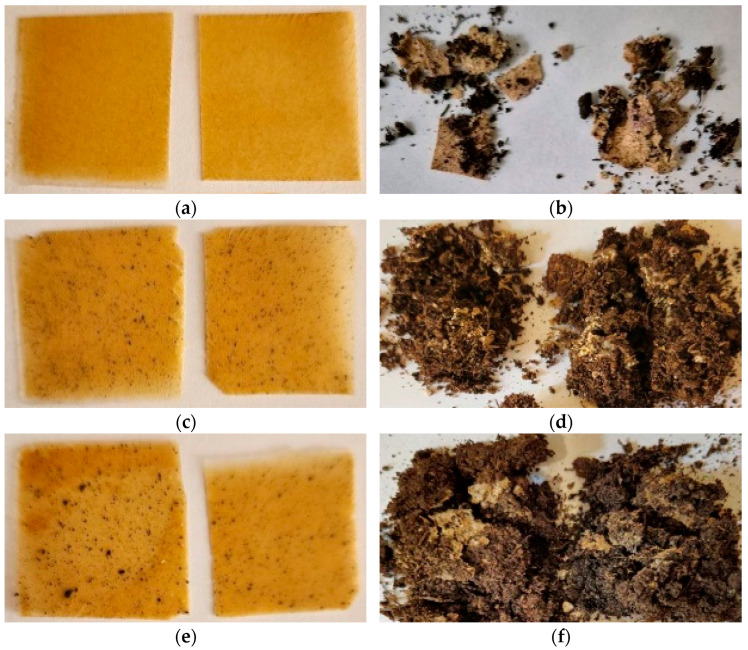
Photographs of films before (**a**,**c**,**e**) and after exposure to soil for 1 month (**b**,**d**,**f**): PLA–PEG_1000_–chitosan (70:10:20 wt%) (**a**,**b**); PLA–PEG_1000_–chitosan + AgNPs (70:10:20 + 0.5 wt%) (**c**,**d**); PLA–PEG_4000_–chitosan + AgNPs (70:10:20 + 0.5 wt%) (**e**,**f**).

In order to evaluate the possibility to biodegrade under natural conditions, the corresponding tests were carried out. According to the technique described, the biocomposite samples were exposed to wet soil for several months with intermittent inspections. Figure 4, Figure 5 and Figure 6 show photos of the samples before and after exposure to soil. The common feature for all systems was that exposure to soil led to drastic changes in the appearance of the films (deformation, integrity, color). In the case of chitosan-based systems with 20 wt% content, the tests lasted only 1 month since the material fractured rapidly (Figure 6). There was no further possibility to collect film pieces to assess the changes in mass.

As was expected, the introduction of polysaccharides led to initiation in biodegradation for the PLA-based systems with AgNPs. The presence of the latter did not affect the process. In the case of biocomposites with starch, the test lasted 6 months since the films became fragile. The maximal mass loss was for the PLA–PEG_1000_–starch + AgNPs (80:10:10 + 0.5 wt%) system and was 29%. After completion, the film observation showed that the biocomposites are covered by dark spots as a result of the vital activity of microorganisms. Similar conclusions can be made after tests with the chitosan-based systems. But in the case of chitosan, the initial films are not transparent, which is why it is difficult to assess the changes that occurred. The maximal mass loss was 12%. Thus, the introduction of biodegradable polysaccharides initiates the biodegradation of the biocomposite as a whole.

### 2.3. Analysis of Changes in Material After Exposure in Soil

**Figure 7:**
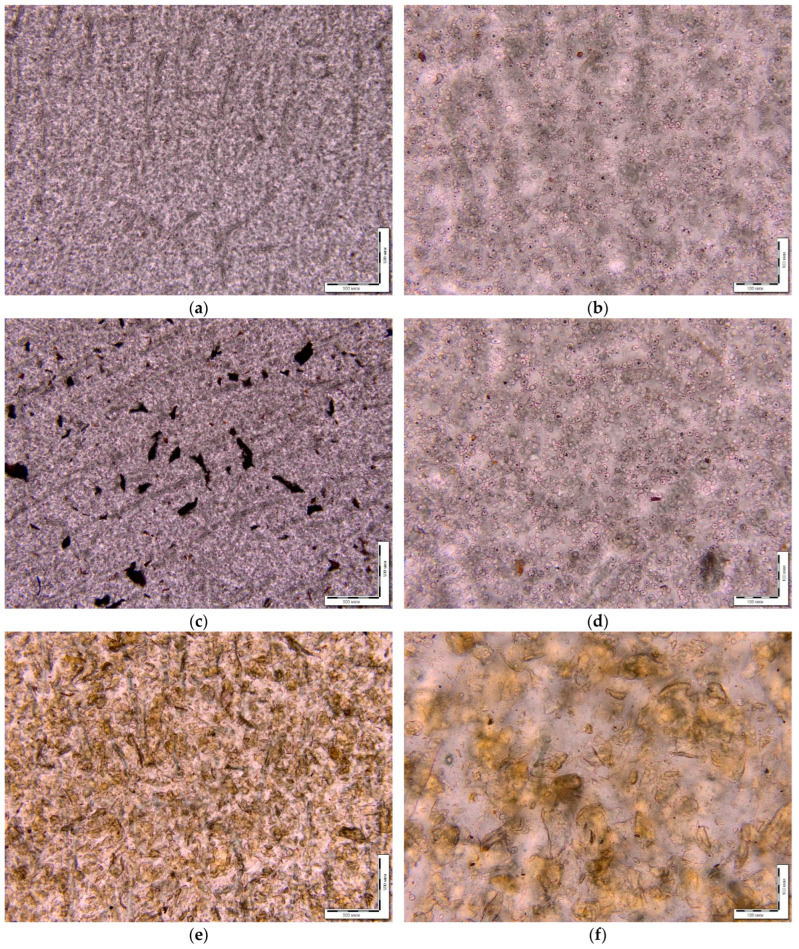

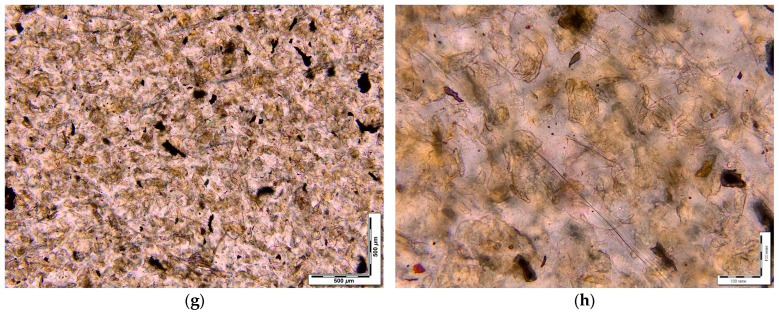
Micrographs of the initial film materials, as seen with transmitted light at different magnifications (×50; ×200): PLA–PEG_1000_–starch (80:10:10 wt%) (**a**,**b**); PLA–PEG_1000_–starch + AgNPs (80:10:10 + 0.5 wt%) (**c**,**d**); PLA–PEG_1000_–chitosan (80:10:10 wt%) (**e**,**f**); PLA–PEG_1000_–chitosan + AgNPs (80:10:10 + 0.5 wt%) (**g**,**h**).

Figure 7 demonstrates the microphotographs of some initial films. According to the microscopy results, the AgNPs particles at a concentration of 0.5 wt% are distributed in the polymer matrix as individual agglomerates. The filler particles (starch and chitosan) are distributed uniformly in the PLA–PEG matrix. Starch particles are regularly spherical with a diameter of 8–15 μm. Chitosan particles are predominantly elliptical in shape with a diameter of 30–200 μm. The critical chitosan content in the composite is 10 wt.%; with a further increase in concentration, the samples are characterized by inhomogeneity.

**Figure 8:**
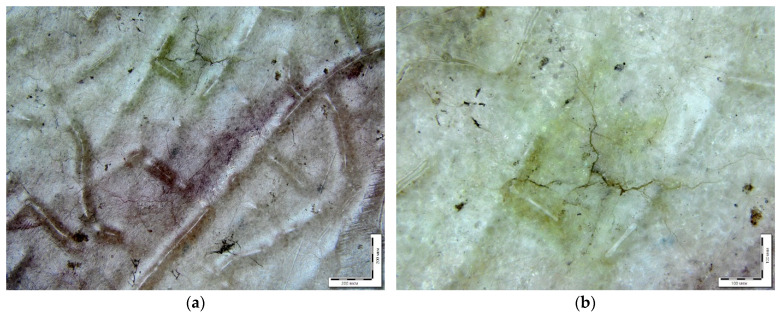

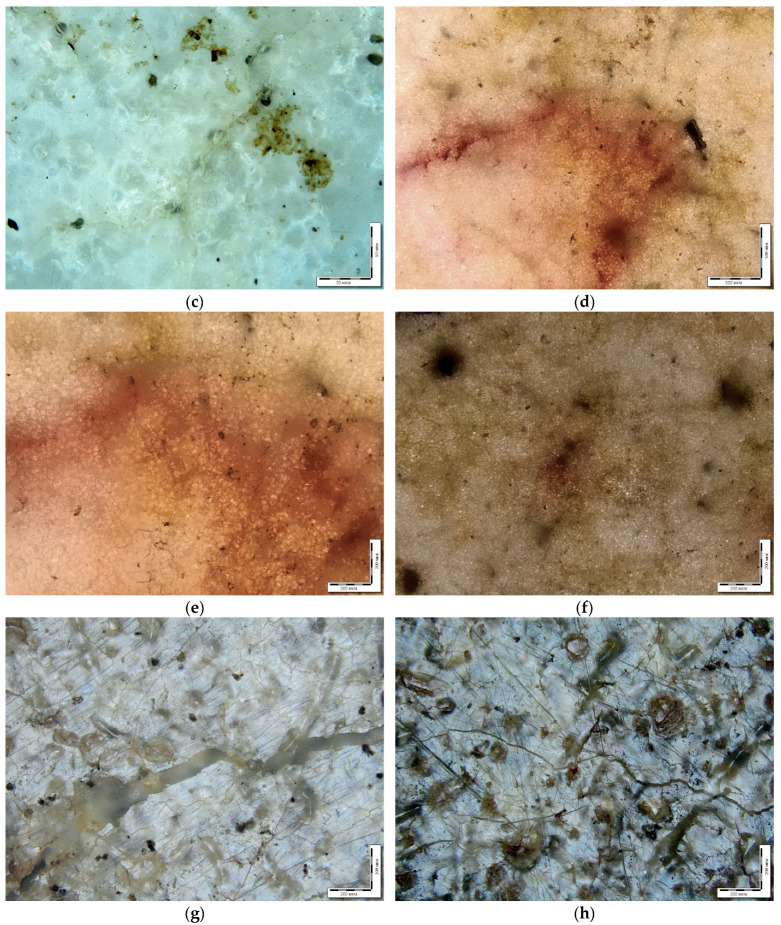

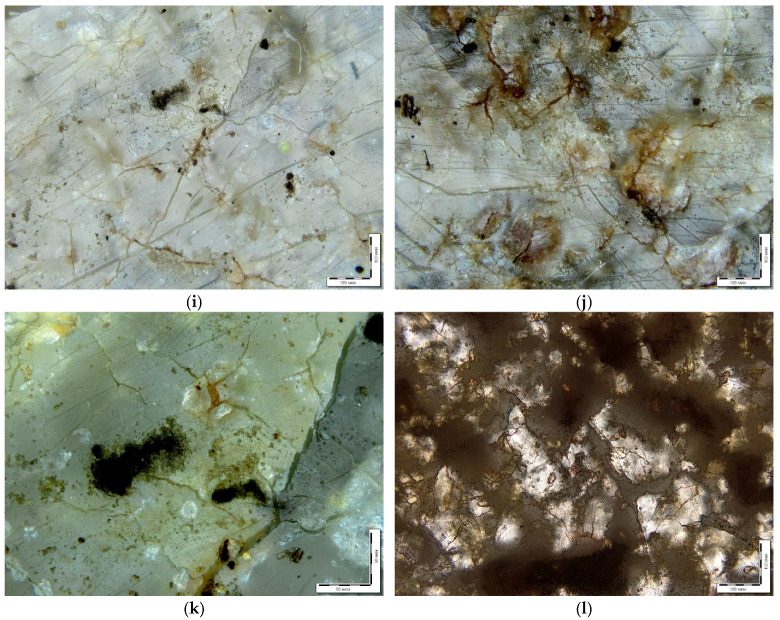
Micrographs of the biocomposite films after several months in soil: PLA–PEG_1000_–starch + AgNPs (80:10:10 + 0.5 wt%) at different magnifications (50, 100, 200, 500): reflected light (**a**–**c**); transmitted light (**d**–**f**); PLA–PEG_1000_–chitosan + AgNPs (80:10:10 + 0.5 wt%): reflected light (**g**–**k**); transmitted light (**l**).

Changes in the appearance of the samples of PLA–PEG_1000_–chitosan + AgNPs (80:10:10 + 0.5 wt%) and PLA–PEG_1000_–starch + AgNPs (80:10:10 + 0.5 wt%) after exposure to soil for 4–6 months are shown in the microphotographs in Figure 8. After the exposure of composite samples with chitosan to soil (4–6 months), numerous cracks and dark areas in the near-surface layers were recorded on the samples using optical microscopy. In some areas, the development of a mycelial network of micromycetes with signs of sporulation was detected. On the composite samples with starch after soil exposure, bright pink-red spots were found within the samples. There are significantly fewer signs of biofouling on the surface than for the composites with chitosan. Samples of the composite with starch (PLA–PEG_1000_–starch + AgNPs (80:10:10 + 0.5 wt%)) after exposure to soil have virtually no mechanical damage in the form of cracks and cavities, i.e., the integrity is preserved.

**Figure 9:**
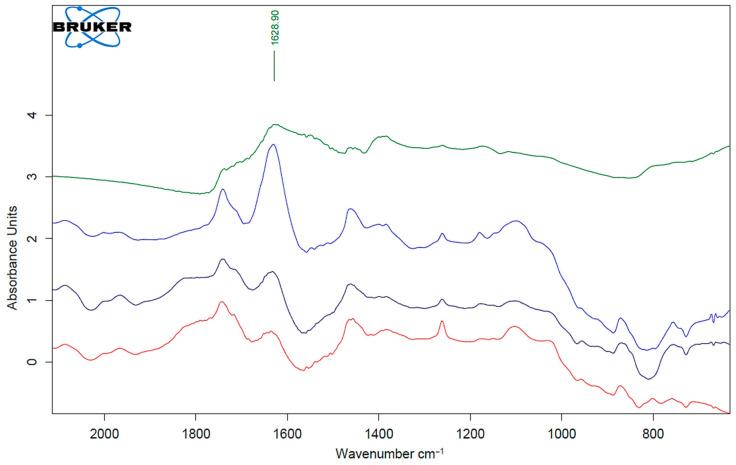
FTIR spectra (transmission mode) of AgNPs particles (green curve), composite sample PLA–PEG1000–starch + AgNPs (80:10:10 + 0.5 wt%) (blue curve), composite sample PLA–PEG1000–starch + AgNPs (80:10:10 + 0.1 wt%) (black curve) and PLA–PEG1000–starch (80:10:10 wt%) (red curve).

The FTIR method allows not only to control the chemical composition of the obtained material but also to track changes occurring in the material under the influence of various factors. The transmission mode was applied to identify AgNPs particles in the initial composite film materials. Figure 9 shows the FTIR spectra of the AgNPs particles and the composites with starch without and with different concentrations of AgNPs particles (0.1 and 0.5 wt%). The silver nanoparticles themselves do not have significant absorption bands in the IR region. The most intensive peak of the AgNP particles was determined at 1630 cm^−1^, which could have been due to the presence of an ethylene maleic acid copolymer. It was this copolymer that was used in the synthesis of the nanosilver particles as an anti-agglomerant [37]. The intensity of the 1630 cm^−1^ band is typical for polyethylene grafted with maleic anhydride (MA-g-PE), indicating the presence of the conjugated carbonyl group (C=O) of the maleic anhydride [38]. According to the obtained results, it was proven that the concentration of silver nanoparticles in the studied materials corresponded to the declared ones.

**Figure 10:**
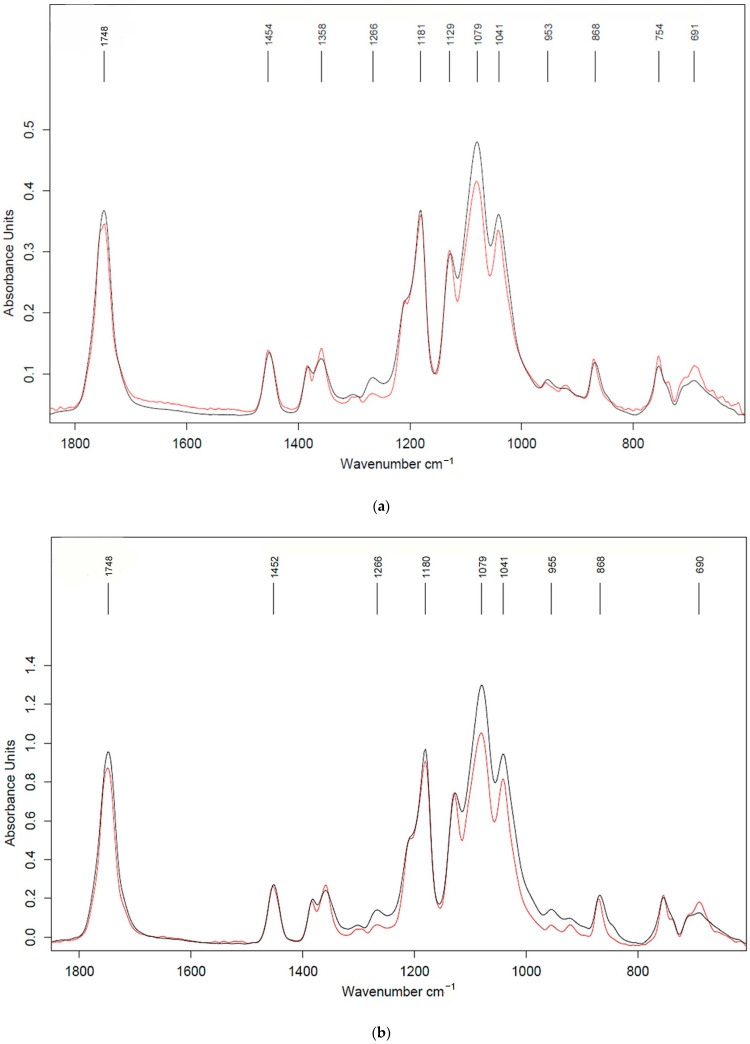
FTIR spectra (ATR mode) of the composites of PLA–PEG_1000_–chitosan + AgNPs (80:10:10 + 0.5 wt%) (**a**) and PLA–PEG_1000_–starch + AgNPs (80:10:10 + 0.5 wt%) (**b**): before (black curve) and after (red curve) exposure in soil test.

Using the FTIR method, changes in the chemical composition that occurred during exposure of the materials to soil were monitored. Since the materials were filled and did not transmit light, surface layer analysis in ATR mode was used. FTIR spectra of the biocomposites before and after the soil test are presented in Figure 10. The results of the FTIR spectroscopy using the ATR method show that the surface layer of the initial composite samples contained neat PLA. Particles of AgNPs and both fillers were encapsulated in the PLA matrix.

After exposure to soil, signs of material degradation were detected (a decrease in the intensity of absorption bands at 1040, 1080, 1266 and 1748 cm^−1^). The decrease in the intensity of these peaks at 1040 and 1080 cm^−1^ bands corresponds to the vibrations of C-O-C groups. These groups are characteristic of PLA and both polysaccharide fillers [39,40]. Thus, the decrease in the absorption intensities of these bands can be explained by the degradation processes of both PLA and the filler.

The decrease in the band intensity at 1266 cm^−1^ for both samples after the soil exposure indicates degradation, specifically of PLA in the composites [41]. This band corresponds to the C-O stretching (ester) vibration in PLA and is a characteristic peak in its FTIR spectrum. The peak at 1748 cm^−1^ is a characteristic and strong signal for PLA, directly corresponding to the stretching of the C=O bond in its ester functional groups. A decrease in this peak indicates a change in the carbonyl functional groups of PLA, which is most often due to polymer degradation through biological breakdown.

**Figure 11:**
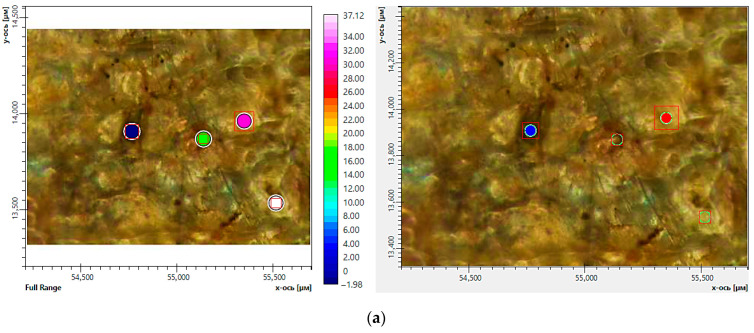

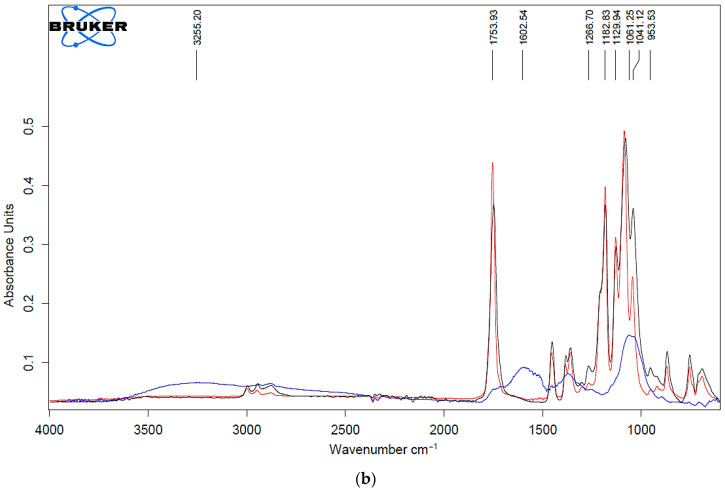
FTIR microscopy of individual areas of the composite of PLA–PEG_1000_–chitosan + AgNPs (80:10:10 + 0.5 wt%): optical imaging mode (**a**) and FTIR spectra (**b**) before (initial sample) (black curve) and after (red and blue curves) exposure to soil (red curve—visually undamaged area; blue curve—dark area of the sample). The red boxes correspond to the spectral aperture. The color of the circles corresponds to the color of the spectra on the graph.

**Figure 12:**
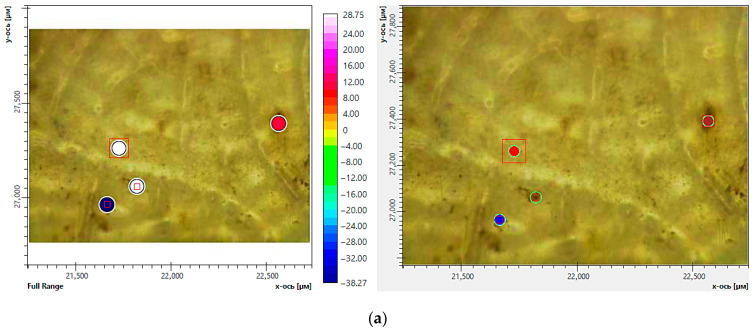

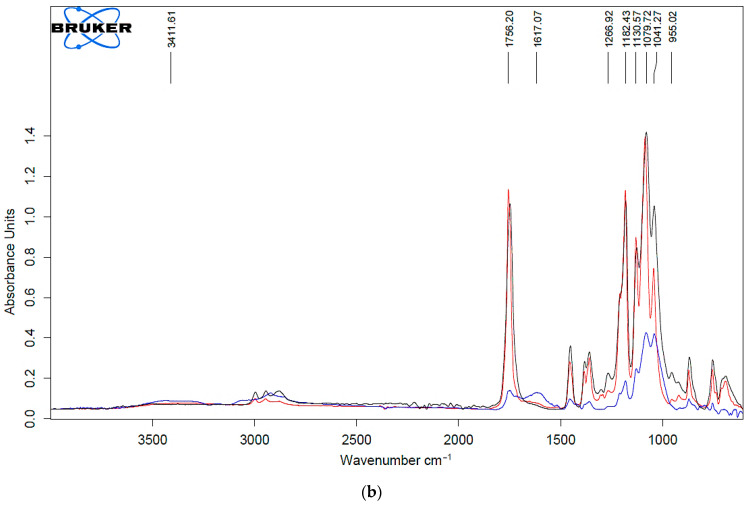
FTIR microscopy of individual areas of the composite of PLA–PEG_1000_–starch + AgNPs (80:10:10 + 0.5 wt%): optical imaging mode (**a**) and FTIR spectra (**b**) before (initial sample) (black curve) and after (red and blue curves) exposure to soil (red curve—visually undamaged area; blue curve—dark area of the sample). The red boxes correspond to the spectral aperture. The color of the circles corresponds to the color of the spectra on the graph.

Using the FTIR microscopy method, the chemical compositions in the individual areas (50–100 μm) of the composite samples were detected after exposure to soil. Figure 11 and Figure 12 demonstrate the areas of FTIR analysis in certain zones and FTIR spectra corresponding to these zones for the composites with chitosan and starch after exposure to soil. It was found that the dark areas on the surface of the materials are of a polysaccharide nature and may be signs of biofouling of the materials by microbial flora (chitin is the main component of the cell wall of micromycete hyphae) [42]. The obtained FTIR spectra of chitin have characteristic absorption bands at 900–1200, 1500–1700 and 3200–3500 cm^−1^ associated with vibrations of various bonds in the chitin molecule, such as C-O, N-H and C-N, O-H, respectively [43]. It is important to note that even the visually undamaged regions were characterized by a decrease in the intensity of absorption bands at 1040 and 1266 cm^−1^ compared with the initial spectra of these composites. The obtained results indicate degradation of PLA in the composites, most likely as a result of enzymatic hydrolysis by soil microbiota. Moreover, the FTIR microscopy demonstrates greater sensitivity in detecting signs of degradation compared with optical microscopy.

**Figure 13:**
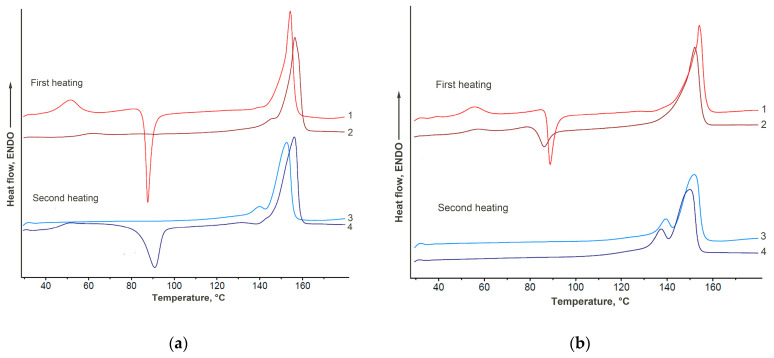
DSC thermograms of PLA–PEG_1000_–starch + AgNPs (80:10:10 + 0.5 wt%) (**a**) and PLA–PEG_1000_–chitosan + AgNPs (80:10:10 + 0.5 wt%) (**b**) biocomposites before (curves 1, 3) and after (curves 2, 4) exposure to soil.

The DSC method was used to analyze the thermophysical properties and crystalline structure of PLA. The behavior of composites based on PLA–PEG_1000_–chitosan + AgNPs (80:10:10 + 0.5 wt%) and PLA–PEG_1000_–starch + AgNPs (80:10:10 + 0.5 wt%) before and after exposure in soil were compared. The total data is presented in Table 2 and Figure 13.

When analyzing the thermophysical properties of composites with chitosan, it was found that during both the first and second heating of samples after exposure to soil, the melting peak of PLA shifts to the region of lower temperatures compared with the original samples (by approximately 2 °C). This fact indicates the degradation of the crystalline regions of PLA during exposure to soil that was shown in other studies [44,45].

During the first heating of the composite sample with starch, a peak of cold crystallization is observed at 87.6 °C. After exposure of the sample to the soil, the peak of cold crystallization is not detected. Moreover, the total enthalpy of melting after the soil experiment slightly increases from 33 to 37 J g^−1^. An increase in the melting temperature from 154.1 to 156.8 °C is also observed. The shown patterns indicate relaxation of the PLA sample in moist soil with subsequent rearrangement of the polymer structure and an increase in its degree of crystallinity. Similar processes occur during annealing of the original sample and during long-term relaxation under normal conditions after the sample preparation process.

When analyzing the behavior of the sample during the second heating, a shift in the PLA melting peak to the region of higher temperatures after exposure to soil is also observed, which indicates an increase in the perfection of the PLA crystallite structure (due to the relaxation process) [46]. However, the low-temperature shoulder of the melting peak significantly decreases, that is, less ordered crystalline formations are destroyed when exposed to soil. This fact is most likely associated with PLA hydrolysis, which at the initial stage of degradation occurs in amorphous and less ordered crystalline regions. A similar effect is shown in other work [47].

The DSC curves obtained during cooling of the samples demonstrate a decrease in the enthalpy of crystallization of PLA after the soil experiment (10.6 J g^−1^) relative to the initial one (30.6 J g^−1^), which also indicates amorphization of the polymer.

### 2.4. Tests on Resistance to Fungi

**Figure 14:**
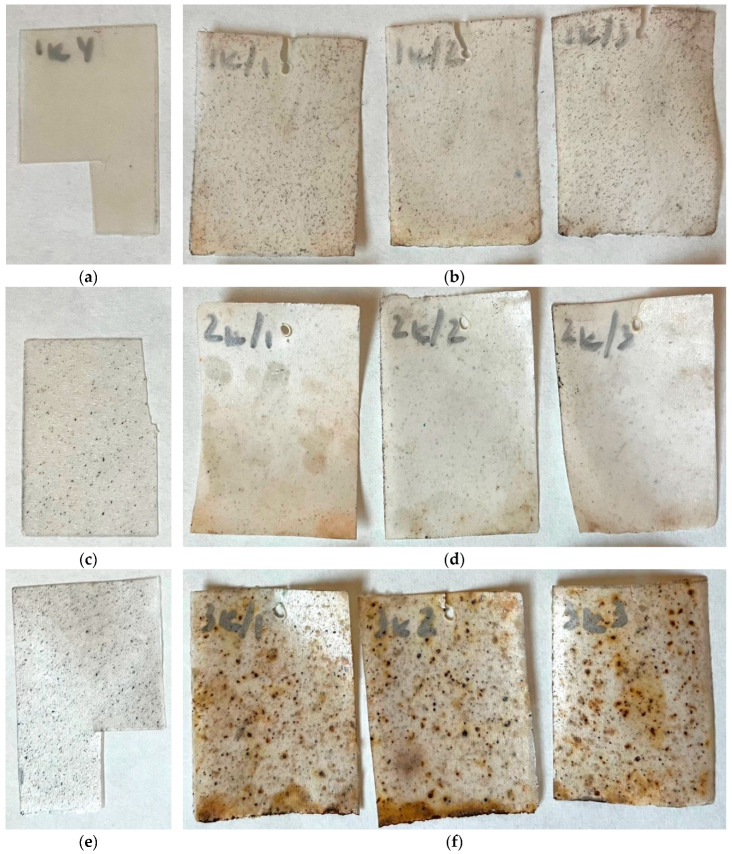
Photographs of control samples (**a**,**c**,**e**) and after 84 days of incubation (**b**,**d**,**f**) of biocomposites: PLA–PEG_1000_–starch (80:10:10 wt%) (**a**,**b**); PLA–PEG_1000_–starch + AgNPs (80:10:10 + 0.1 wt%) (**c**,**d**); PLA–PEG_1000_–starch + AgNPs (80:10:10 + 0.5 wt%) (**e**,**f**).

**Figure 15:**
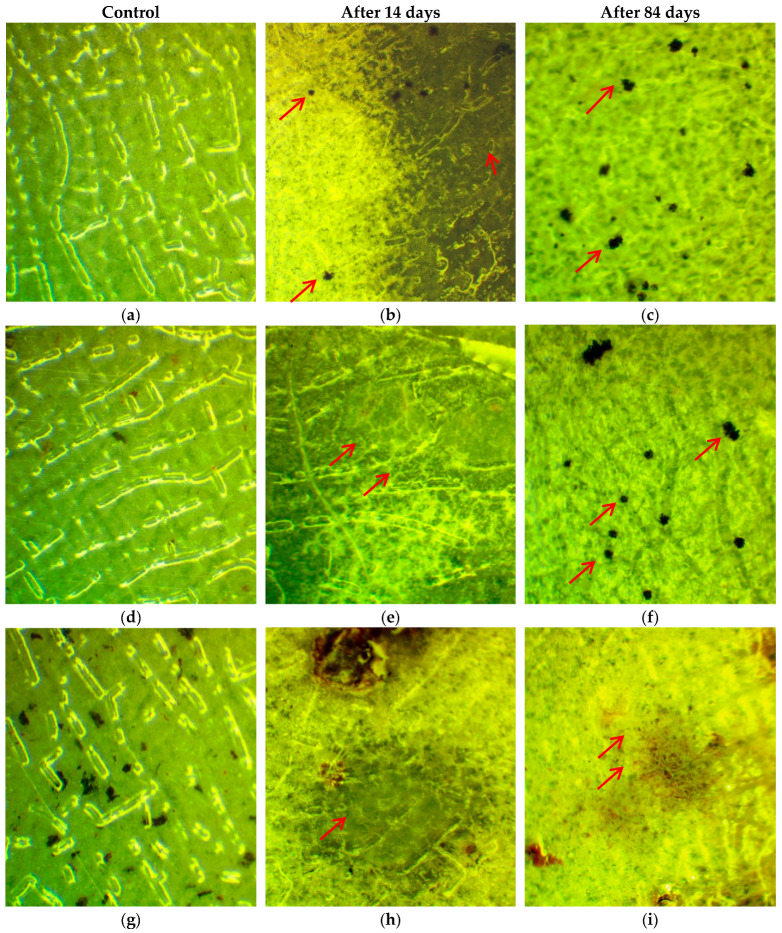
Photographs of control samples and after 14 and 84 days of incubation of biocomposites: PLA–PEG_1000_–starch (80:10:10 wt%) (**a**–**c**); PLA–PEG_1000_–starch + AgNPs (80:10:10 + 0.1 wt%) (**d**–**f**); PLA–PEG_1000_–starch + AgNPs (80:10:10 + 0.5 wt%) (**g**–**i**) (×50).

**Figure 16:**
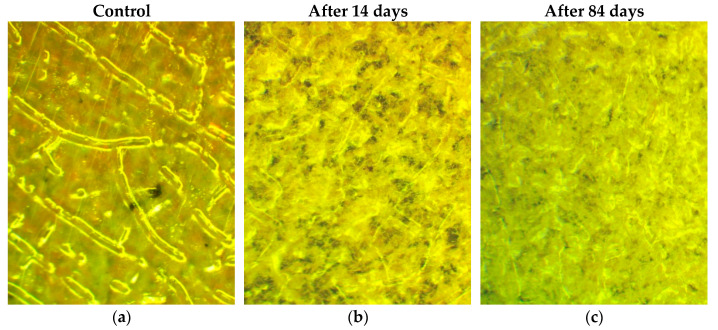

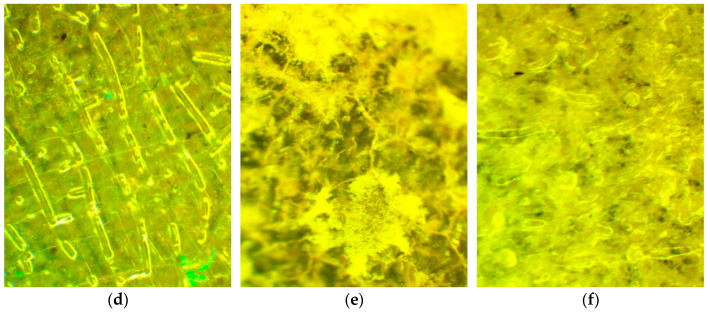
Photographs of control samples and after 14 and 84 days of incubation of biocomposites: PLA–PEG_1000_–chitosan (80:10:10 wt%) (**a**–**c**); PLA–PEG_1000_–chitosan + AgNPs (80:10:10 + 0.1 wt%) (**d**–**f**) (×50).

In order to evaluate the ability to biodegrade, the tests on fungus resistance were carried out according to a State Standard. The mold fungi used are traditional for the region of the experiments performed. The biocomposites based on PLA with PEG containing starch/chitosan with and without silver nanoparticles were tested for a maximal 84 days. The results are presented in detail in Table 3 and Figure 14, Figure 15 and Figure 16. Since the intensity of fungal growth on the samples was evaluated according to a six-point scale, the corresponding points for the studied biocomposites are presented in Table 3 depending on the day of the test.

All biocomposites under investigation demonstrated a fairly high biodegradability under the action of mycelial fungi, while the addition of AgNPs somewhat reduced the intensity of fungal development under the given conditions. On the films without AgNPs, the growth of fungi, barely visible to the naked eye, was noted after 2 weeks of observation, and by the end of the experiment (84 days), the development of fungi covering 100% of the surface of the plates was clearly visible (Table 3). At the same time, it was easy to determine the dominant biodestructor fungus *Aspergillus brasiliensis*, the characteristic spore formation of which (conidia collected in black heads) is clearly visible to the naked eye and confirmed by microscopy (Figure 15a,b and Figure 16a–c).

Introduction of 0.1 wt% AgNPs into the biocomposites somewhat reduced the intensity of fungal development; during the first 28 days of observation, growth was visible only under microscopy, while sporulation was absent; by the 56th day, sporulation of *Aspergillus brasiliensis* was detected with the naked eye, and by the end of the observation, growth was clearly visible, covering about 20% of the surface of the films (Figure 14c,d and Figure 15d–f).

Increasing the concentration of AgNPs in the biocomposites up to 0.5 wt% had an even greater effect on the intensity of fungus growth. Barely noticeable growth, visible to the naked eye, was noted only toward the end of the observation. At the same time, sporulation was almost not observed; microscopy of the samples showed the presence of developed microcolonies formed by sterile mycelium (Figure 15g–i). Using the spreading technique, we found that in addition to *Aspergillus brasiliensis*, which is typical for these systems, another representative of the *Aspergillus* genus, *Aspergillus sojae*, actively developed.

All biocomposites under investigation completely lost their transparency by the end of the observations, regardless of the presence or absence of AgNPs. It is interesting to note that the biocomposite containing 0.5 wt% AgNPs changed its color significantly (due to the appearance of “rusty-colored” inclusions in the lower part of the plates) until the color completely changed to dark brown (Figure 14e,f). In addition, the experimental conditions (relative air humidity over 90%) led to “flowing/sliding” of the brown-colored mass already on the 14th day of the experiment.

In should be noted that the changes in mass loss were impossible to detect since the materials lost their integrity during the test. It is interesting to note that water droplets formed on all the samples, especially in the first weeks of the experiment, and most actively on the PLA–PEG_1000_–starch + AgNPs (80:10:10 + 0.1 wt%) biocomposite, which led to an uneven loss of transparency of the material. No such phenomena were observed on systems of different composition under the same experimental conditions.

**Figure 17:**
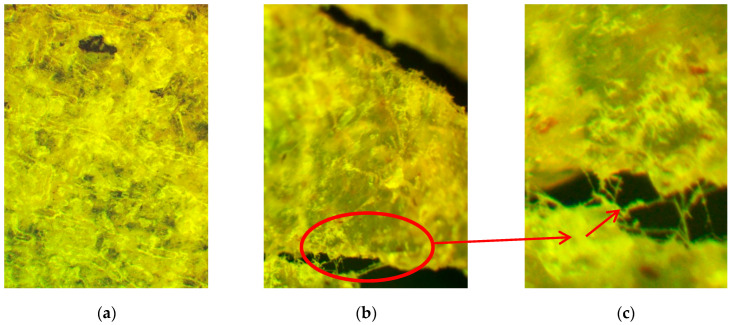

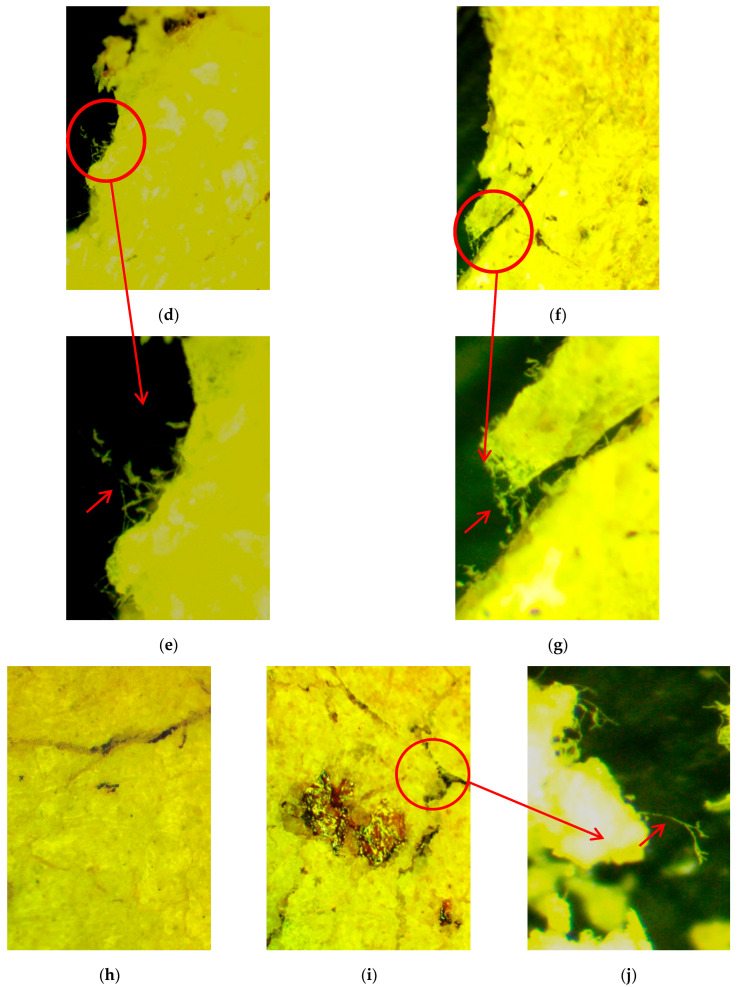
Photographs of samples after 14 (**a**,**d**,**e**,**h**) and 84 days of incubation (**b**,**c**,**f**,**g**,**i**,**j**) of biocomposites: PLA–PEG_1000_–chitosan + AgNPs (80:10:10 + 0.5 wt%) (**a**–**c**); PLA–PEG_1000_–chitosan (70:10:20 wt%) (**d**–**g**); PLA–PEG_1000_–chitosan + AgNPs (70:10:20 + 0.5 wt%) (**h**–**j**) (×50).

**Figure 18:**
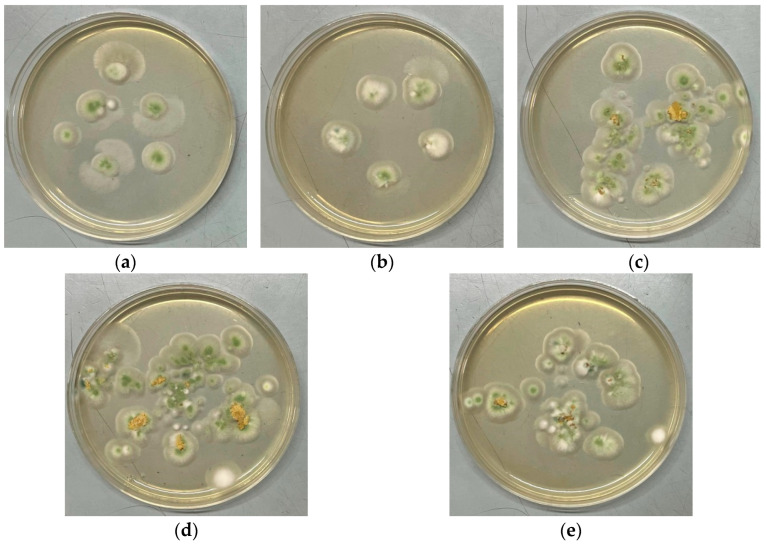
Spreading technique to define dominating destructor of biocomposites: PLA–PEG_1000_–chitosan (80:10:10 wt%) (**a**); PLA–PEG_1000_–chitosan + AgNPs (80:10:10 + 0.1 wt%) (**b**); PLA–PEG_1000_–chitosan + AgNPs (80:10:10 + 0.5 wt%) (**c**); PLA–PEG_1000_–chitosan (70:10:20 wt%) (**d**); PLA–PEG_1000_–chitosan + AgNPs (70:10:20 + 0.5 wt%) (**e**).

In the case of biocomposites with chitosan, different compositions were used, including the system that contained up to 20 wt% of chitosan (Table 3, Figure 16, Figure 17 and Figure 18). The latter content of chitosan led to “soaking” of the plates immediately after infection by the mold fungus spore suspension and placement under wet conditions; as a result, some parts of the plates fell into water. In general, the biocomposites with chitosan are less elastic than similar systems with starch. The estimation of the fungus resistance for all biocomposites with chitosan at different periods of the test is presented in Table 3.

The biocomposites with chitosan under investigation demonstrated sufficiently high biodegradability under the influence of mycelial fungi under the given incubation conditions, while the addition of AgNPs did not reduce the intensity of fungal development.

All samples lost their transparency in just 7 days of incubation; microscopy clearly showed a significant increase in the volume of the material (swelling), which was more noticeable in the PLA–PEG_1000_–chitosan (70:10:20 wt%) and PLA–PEG_1000_–chitosan + AgNPs (70:10:20 + 0.5 wt%) biocomposites.

As in the case of the biocomposites with starch, the changes in mass loss were impossible to detect since the materials lost their integrity during the test. The coloring of the samples greatly masked the existing fungal growth. On all the plates, it could be seen only in places where the integrity of the material was compromised (Figure 16 and Figure 17), where the fungal mycelium and sporulation characteristic of *Penicillium* fungi were clearly visible. It is clearly visible that the mycelium permeates the entire thickness of the material, which may be associated with its significant swelling and, as a result, a decrease in the density. It can be assumed that the growth of fungi covers the entire surface of the plates in an even layer, but it is impossible to confirm this with the naked eye.

The direct plating on an agarized nutrient medium confirmed the assumption that mycelial fungi actively develop on the biocomposites containing chitosan. Scraping the fungi from the surface of the plates made it possible to identify the dominant destructor among the test organisms used, namely, *Penicillium chrysogenum* VKM F-245 (Figure 18).

It is obvious that addition of AgNPs into the biocomposites did not have a global effect on the biodegradation. We observed only a slight decrease in fungal development in the presence of starch in the biopolymers, and the more AgNPs that were added, the greater the decrease. This can be explained since there is evidence that silver ions can affect the alpha-amylase enzyme, reducing its activity [48,49].

Despite this, all the samples under investigation with starch were subjected to active destruction by fungi. Already after 14 days of observation, with a slight increase in the area of fungal damage, microscopy showed the presence of sporulation, which is characteristic of fungi of the genus *Aspergillus*, which is known as one of the most active biodestructors that destroy a wide range of polymeric materials. These fungi have a diverse set of constitutive and adaptive enzymes capable of enzymatically cleaving bonds in polymer chains. In addition, they produce bioactive metabolites, for example, during the growth process, they form various organic acids [50], which also increases their destructive activity. Further observations showed an increase in the colonization area and the intensity of fungal development in all samples.

Experiments with biocomposites containing chitosan have shown that despite the fact that chitosan itself has biocidal properties, compositions based on it are biodegradable regardless of AgNPs. Increasing the chitosan content in the samples led to its faster destruction, including mechanical destruction due to the penetration of mycelium into the volume of the samples. The dominant destructor in this case is *Penicillium chrysogenum*, whose enzymatic system is extremely developed, and the number of metabolites produced is so great that this organism is currently being developed as an “industrial platform for heterologous production of metabolites” [51].

Thus, the tests on fungus resistance showed that biocidal additives such as AgNPs in PLA-based biocomposites with polysaccharides do not inhibit the development of mycelial fungi–biodestructors. And the increased amount of chitosan in the films contributed to their more active destruction by the end of the observation period.

### 2.5. Tests on Antibacterial Activity

With the aim to assess the antibacterial activity of the films obtained, the samples were infected with a set of different microorganisms (yeasts and Gram-positive and Gram-negative bacteria). As in a previous investigation [36], there was no zone of clearance around the film samples with any of the microorganisms. The results of the experiment are presented in Table 4. There was no growth of *E. coli*, *M. luteus* and *B. subtilis* under the film samples, which indicates a complete inhibitory effect for these microorganisms. In the case of *C. sporogenes*, weak growth was observed only under the films without AgNPs, while the introduction of the latter led to full inhibition of its growth. At the same time, both compositions with starch weakly inhibited the growth of yeasts.

The inhibiting ability of samples with chitosan was also estimated by the growth intensity around and under the films. But in contrast to starch-based composites, the films with chitosan were not opaque. Also, the antibacterial activity of chitosan known from the literature (for example [52,53]) should be taken into account. Nevertheless, after removing the sample plates from the agar surface, no growth of *G. auringiensis*, *E. coli*, *M. luteus* and *B. subtilis* was observed (Table 4).

In view of the data obtained for biocomposites with chitosan when evaluating agar under film, the inhibitory effect of the film samples was tested when culturing with microorganisms in a liquid medium. The inhibition degree of test cultures by the samples was assessed by the degrees in optical density (OD_600_) compared with control culture without biocomposite films (Table 5). *M. luteus* showed the greatest sensitivity to the films under investigation in a liquid nutrient medium, and its growth was completely inhibited. The growth of the yeast *G. auringiensis* was significantly inhibited by the sample with AgNPs, while the sample without silver suppressed the growth of the yeast to a lesser extent. Both studied samples slightly inhibited the growth of *B. subtilis*. The presence of PLA-based biocomposites with chitosan with/without AgNPs in the medium did not affect the growth of *E. coli* and *C. sporogenes*. Probably, a larger amount of biocomposite material is required to inhibit the growth of these bacteria in a liquid medium.

## 3. Materials and Methods

### 3.1. Materials

Initial components for synthesis of silver nanoparticles (AgNPs): poly(ethylene-alt-maleic anhydride) (“Monsanto”, St. Louis, MO, USA) (average molecular weight M = 25,000), NaBH_4_ (“Sigma-Aldrich”, Darmstadt, Germany), and AgNO_3_ and NaOH (both of analytical grade, “Reahim”, Moscow, Russia) were used without purification. Before use, the copolymer was hydrolyzed to the corresponding copolymers of maleic acid (EM) by dissolving in deionized water followed by lyophilization (−55 °C, 0.05 mbar) [36].

PLA 4043D (“NatureWorks”, Plymouth, MN, USA) with *T_m_* = 145–160 °C, density 1.24 g/cm^3^ and MFI = 6.0 g/10 min (210 °C under 2.16 kg loads); PEGs (Sigma, Steinheim, Germany) of different molecular weights (1000, 4000); potato starch (“Slavnaya Trapeza Kholding”, Tovarkovo village, Kaluga Region, Russia); and chitosan (“Bioprogress” CJSC, Biokombinat village, Moscow Region, Russia) were used for the biocomposites.

### 3.2. Synthesis of AgNPs

According to a technique [54], the silver hydrosol EM/Ag^0^ was prepared by the borohydride reduction of silver salt in the presence of EM. First, AgNO_3_, NaBH_4_ and a polymer stabilizer (EM) were dissolved in deionized water separately. In order to obtain the aqueous solutions of EM, the solid copolymer was dissolved in water at room temperature. Then a 1 M solution of NaOH was added for a pH = 6 adjustment (using a Fisher Scientific 300 403.1 pH-meter (Waltham, MA, USA)). Finally, the solutions were vigorously stirred during and after (for 30 min) addition of 2-fold molar excess of aqueous NaBH_4_ at room temperature. The reaction mixture was allowed to stand (24 h, 200 °C). A dried EM/Ag^0^ sample was obtained after ultrafiltration (cutoff 3.5 kDa) and freeze-drying (−55 °C, 0.05 mbar).

### 3.3. Production of PLA–PEG–Polysaccharide + AgNPs Composites

The mixing of components was carried out in a twin-screw microextruder HAAKE Rheomex Minilab II (Thermo Scientific, Waltham, MA, USA) under the joint action of shear deformations and high temperatures. PLA was melted first, then PEG with polysaccharide or PEG with silver nanoparticles (AgNPs) and polysaccharide (starch, chitosan) were introduced into the PLA melt; the components were mixed at 170 °C for 10 min and 50 rpm. After the lapse of time, the mixtures were extruded through a flat slit.

### 3.4. Characterizations

#### 3.4.1. Pressing and Mechanical Characteristics

The composites were pressed on a Carver CH 4386.4010 (Wabash, IN, USA) laboratory press at 170 °C and 10 MPa for 10 min followed by cooling under pressure at a rate of ~15 °C/min. The films obtained with a thickness of ~0.3 mm were used in the following tests.

Mechanical characteristics were obtained using an Instron-6669 (Norwood, MA, USA) universal testing machine in a stretching mode at a rate of the upper traverse movement of 1 mm/min and at room temperature. The elastic modulus (*E*), ultimate tensile stress (*σ_b_*), and relative elongation at break (*ε_b_*) were calculated from the stress (*σ*)–strain (*ε*) diagrams. The results were averaged for 4–5 samples. The errors of *E* and *σ_b_* did not exceed 10%, and the error of *ε_b_* was 5%.

#### 3.4.2. Morphology

The samples were cut as 5 × 5 mm sheets and were placed onto electro-conductive carbon tape adhered to an SEM sample holder. The cross-sections of samples were obtained by liquid nitrogen cooling followed by brittle fracture. A gold layer of 10 nm thickness was deposited on the sample surface inside spatter coater Q 150R ES (Quorum, London, UK) followed by sample loading inside a chamber of Prisma E (Thermo Fisher Scientific, Waltham, MA, USA) scanning electron microscope. SEM imaging was performed in secondary electron mode at low voltage settings (0.5–1.5 kV) to demonstrate surface features of each sample.

#### 3.4.3. Thermal Properties

Differential scanning calorimetry (DSC) was performed using a differential scanning calorimeter DSC 214 Polyma NETZSCH-Gerätebau GmbH (Selb, Germany). Temperature scanning program was in the range of 20 to 190 °C: 1st heating, 1st cooling and 2nd heating. Measurements were carried out in closed aluminum pans (Concavus NETZSCH-Gerätebau GmbH (Selb, Germany)) in an air environment at a heating rate of 10 °C/min; the sample weight was 5–10 mg. The experiment was performed with two repetitions (averaged data is presented in Table 2). Thermograms were processed using Proteus NETZSCH software (version 8.0.1).

#### 3.4.4. Resistance to Fungi

Fungus resistance was carried out according to a State Standard (GOST) 9.049-91 [55] under conditions of fungal growth only due to nutrients in the samples (method 1). Test specimens of biocomposites (plates 40 × 60 mm in size, 3 replicates) were exposed to a mixed suspension of fungal spores in water in the presence of ≥90% relative humidity and a temperature of 29 ± 2 °C. Fungal test organisms were taken from the All-Russian Collection of Microorganisms (VKM) (Table 6).

The concentration of each strain spore used in the test in the suspension was 1–2 mln/cm^3^. The tests lasted for 84 days with intermittent inspections at 7, 14, 21, 28 and 56 days. The fungus resistance in terms of the intensity of fungus growth on the samples was evaluated according to a six-point scale. Furthermore, the tests assessed the change in appearance of the samples.

To identify the dominant biodestructors, the method of seeding on an agarized nutrient medium CZA (Chapek agar) was used. At the end of the testing, a scraping was made from the surface of each plate with a sterile metal needle, and the selected material was transferred to the surface of the medium in a Petri dish and cultivated for 7 days at 30 °C. The developed fungi were identified based on phenotypic characteristics.

#### 3.4.5. Antibacterial Activity

In order to evaluate the antibacterial activity of the biocomposites, the set of microorganisms from VKM was used (Table 7). Microorganisms were applied to the surface of the solid medium in the amount of 100 μL of night culture on a Petri dish. Medium composition (g/L) for *B. subtilis*, *E. coli* and *M. luteus*: aminopeptide 60 mL; trypton 5.0 g; yeast extract 1.0 g; soybean extract 30 mL; bacto-agar 15.0 g; final pH 7.2. Medium composition for *G. auringiensis*: malt extract 15.0; dextrin 2.75; peptone 0.75; ammonium chloride 1.0; dipotassium phosphate 1.0; D-maltose 12.75; bacto-agar 15; final pH 5.4. Medium composition for *C. sporogenes*: trypticase 5.0; peptone 5.0; yeast extract 10.0; salt solution 40.0 mL; Na-resazurin (0.1% *w*/*v*) 0.5 mL; L-cysteine-HCl 0.5; Na_2_CO_3_ 2.5; D-glucose 5.0; bacto-agar 15.0. Salt solution (g/L): CaCl_2_ × 2H_2_O 0.25; MgSO_4_ × 7H_2_O 0.5; K_2_HPO_4_ 1.0; KH_2_PO_4_ 1.0; NaHCO_3_ 10.0; NaCl 2.0. Then, the test specimens of biocomposites with a size of approximately 1.0 × 1.0 cm, pre-treated in 80% ethanol within 24 h and washed by sterile distilled water was placed on the surface of the agar with 3 replicates. Cultivation was carried out at 30 °C for 120 h in two replicates. *B. subtilis*, *E. coli*, *M. luteus* and *G. auringiensis* were cultivated aerobically, but *C. sporogenes* was grown in an anaerobic jar under 100% nitrogen atmosphere. The inhibitory capacity of the film samples was assessed by the presence of bacterial and yeast growth around the film samples and under one.

Along with this test, the inhibitory capacity was assessed in liquid media. The 1.0 × 1.0 cm test specimens of biocomposites were placed in a test tube with a nutrient medium and sterilized at 110 °C for 30 min. Then, 100 μL of the overnight culture was added to the test tube and cultivated on a rotary shaker at 200 rpm for 24 h. The degree of inhibition of the test cultures by samples was assessed by the change in optical density (OD_600_) compared with control test tubes without samples. All tests were performed in triplicate.

#### 3.4.6. IR-Spectroscopy

Fourier transform infrared spectroscopy (FTIR) was conducted by means of a Lumos Bruker FTIR microscope (Germany):

– By the attenuated total internal reflection method (diamond crystal (ATR platinum Diamond));
– In transmission mode in a potassium bromide matrix;
– In FTIR microscope mode (ATR method, germanium crystal, shooting aperture 50–100 μm).

The spectra were recorded at a temperature of 23 ± 2 °C in the mid-infrared spectrum in the wavenumber range of 650–4600 cm^−1^. The spectra were processed using Bruker OPUS software (version 8.1).

#### 3.4.7. Soil Tests

The tests on biodegradability of biocomposites were conducted via imitating the environmental conditions according to a technique based on an ASTM D5988-12 standard [56]. For this aim, the samples under investigation were placed into a container with wet soil (pH 6–7) designated for plant growing (OOO “Russkaya Torfyanaya Kompaniya”, Krasnogorsk, Russia). The soil composition: high-moor peat, agroperlite, biohumus, dolomite dust, complex liquid fertilizer and natural zeolite. Containers were incubated at room temperature for 6–8 months. The biodegradation rate was estimated by the mass loss of samples determined at certain time periods.

#### 3.4.8. Optical Microscopy

Photography was conducted using an Olympus BX3M-PSLED microscope (Tokyo, Japan) and an Olympus Sort Imaging Solutions LC30 camera. Shooting used transmitted light at magnifications of 50, 100, 200 and 500× (MPlanFLN objectives). Processing of microphotographs was performed using Olympus Steam Basic software (version 2.4.4).

## 4. Conclusions

Under conditions of shear deformations in a twin-screw extruder, PLA-based biocomposites with PEG and AgNPs with polysaccharides (starch and chitosan) introduced were obtained. Analysis of the mechanical characteristics of the systems under investigation showed that the introduction of polysaccharides led to a significant decrease in the elastic modulus and tensile strength, while the elongation at break remained almost unchanged. Such an effect can be dictated by both the nature of polysaccharides (rigid polymers) and content of AgNPs. Biodegradability of the systems was estimated by the evaluation of mass loss after exposure in soil. In common, the results indicate that the presence of natural polysaccharides intensified the biodegradation in soil despite the AgNP availability. For all the samples, the exposition in soil led to drastic changes in the appearance of films, namely, deformation, integrity and color. The maximal mass loss was for the PLA–PEG_1000_–starch + AgNPs (80:10:10 + 0.5 wt%) system and was 29%. Analyses of the systems before and after soil exposure were carried out using optical microscopy, DSC and FTIR spectroscopy methods. According to photographs from the optical microscopy, the maximal allowed content of AgNPs was 0.1 wt%. Thermal analysis showed that the low-temperature shoulder of the melting peak significantly decreased, that is, less ordered crystalline formations were destroyed when exposed to soil. This fact is most likely associated with PLA hydrolysis, which in the initial stage of degradation occured in amorphous and less ordered crystalline regions. At the same time, the same conclusion was formed regarding the spectral analysis: the intensity of the characteristic absorption bands of PLA decreased. Analysis of the dark areas on the surface of the materials are of a polysaccharide nature and may be signs of biofouling of the materials by microbial flora. The detailed analysis of the results of tests on fungus resistance showed that intensity of fungus growth for samples with starch achieved the maximal 5 points, while for chitosan-based systems, this value was 3 points. Despite this estimate, such biocidal additives as AgNPs in PLA-based biocomposites with polysaccharides do not inhibit the development of mycelial fungi–biodestructors. The antibacterial activity evaluated using different microorganisms showed that these systems actively inhibited the development of the latter. In the case of chitosan in liquid medium, it was demonstrated that the presence of PLA-based biocomposites with chitosan with/without AgNPs in the medium did not affect the growth of *E. coli* and *C. sporogenes*. Probably, a larger amount of biocomposite material is required to inhibit the growth of these bacteria. Analysis of the effect of AgNPs on their characteristics showed that the biocomposites developed meet the modern challenges of polymer chemistry for obtaining multifunctional materials used in various fields, such as packaging.

## Figures and Tables

**Table 1:** Mechanical characteristics of pure PLA and biocomposites.

Compositions	*E*, MPa	*σ_b_*, MPa	*ԑ_b_*, %
PLA (100 wt%)	3322.5	41.3	2.9
PLA–PEG_1000_–starch (80:10:10 wt%)	692.5	9.5	2.3
PLA–PEG_1000_–starch + AgNPs (80:10:10 + 0.1 wt%)	1100	7.1	2.5
PLA–PEG_1000_–starch + AgNPs (80:10:10 + 0.5 wt%)	1392.5	19.3	1.8

**Table 2:** Thermophysical properties of the composite samples of PLA–PEG_1000_–starch + AgNPs (80:10:10 + 0.5 wt%) and PLA–PEG_1000_–chitosan + AgNPs (80:10:10 + 0.5 wt%) (ND—not detected, NA—not applicable).

Experiment in Soil	Glass Transition Temperature (°C)	Crystallization		Melting	
		Onset Temperature (°C)	Enthalpy (J g^−1^)	Onset Temperature (°C)	Enthalpy (J g^−1^)
PLA–PEG_1000_–Starch + AgNPs (80:10:10 + 0.5 wt%)					
First heating					
Before	47.1	87.6	16.3	154.1	33.4
After	58.7	ND	ND	156.8	37.7
Cooling					
Before	ND	87.9	30.6	NA	NA
After	43.0	88.2	10.6	NA	NA
Second heating					
Before	ND	ND	ND	153.4	36.0
After	47.8	89.6	15.7	155.9	38.5
PLA–PEG_1000_–Chitosan + AgNPs (80:10:10 + 0.5 wt%)					
First heating					
Before	51.0	88.8	9.0	154.3	32.3
After	54.2	86.6	5.0	152.0	35.5
Cooling					
Before	ND	96.4	31.8	NA	NA
After	ND	95.7	34.1	NA	NA
Second heating					
Before	ND	ND	ND	151.8	35.0
After	ND	ND	ND	150.2	35.3

**Table 3:** Assessment of fungal growth on test specimens (in points *) depending on day of test.

Biocomposites	Day of Test					
	7	14	21	28	56	84
PLA–PEG_1000_–starch (80:10:10 wt%)	2	3	3	3	5	5
PLA–PEG_1000_–starch + AgNPs (80:10:10 + 0.1 wt%)	1	2	2	2	3	4
PLA–PEG_1000_–starch + AgNPs (80:10:10 + 0.5 wt%)	1	2	2	2	2	3
PLA–PEG_1000_–chitosan (80:10:10 wt%)	0	2	2	3	3	3
PLA–PEG_1000_–chitosan + AgNPs (80:10:10 + 0.1 wt%)	0	2	2	3	3	3
PLA–PEG_1000_–chitosan + AgNPs (80:10:10 + 0.5 wt%)	1	2	3	3	3	3
PLA–PEG_1000_–chitosan (70:10:20 wt%)	1	2	3	3	3	3
PLA–PEG_1000_–chitosan + AgNPs (70:10:20 + 0.5 wt%)	1	2	3	3	3	3

* 0—no growth apparent under the microscope; 1—no growth visible to the naked eye but germinated spores and poor mycelium visible under the microscope; 2—no growth visible to the naked eye but advanced mycelium and (or) sporulation visible under the microscope; 3—growth insignificantly visible to the naked eye but clearly visible under the microscope; 4—growth visible to the naked eye, covering up to 25% of the test surface; 5—growth visible to the naked eye, covering more than 25% of the test surface.

**Table 4:** Growth of test cultures under the film samples (NA—not applicable).

Biocomposites (wt%)	*G. auringiensis*	*E. coli*	*M. luteus*	*B. subtilis*	*C. sporogenes*
PLA–PEG_1000_–starch (80:10:10 wt%)	Weak growth	No growth	No growth	No growth	Weak growth
PLA–PEG_1000_–starch + AgNPs (80:10:10 + 0.5 wt%)	Weak growth	No growth	No growth	No growth	No growth
PLA–PEG_1000_–chitosan (80:10:10 wt%)	No growth	No growth	No growth	No growth	N/A
PLA–PEG_1000_–chitosan + AgNPs (80:10:10 + 0.5 wt%)	No growth	No growth	No growth	No growth	N/A

**Table 5:** Results of measuring the optical density of test cultures during cultivation with sample plates in a liquid medium.

Coating Sample	*G. auringiensis*	*E. coli*	*M. luteus*	*B. subtilis*	*C. sporogenes*
Control (without test film)	5.36 ± 0.12	2.10 ± 0.04	4.85 ± 0.04	2.91 ± 0.15	1.97 ± 0.04
PLA–PEG_1000_–chitosan (80:10:10 wt%)	4.62 ± 0.12	2.14 ± 0.03	0.137 ± 0.001	2.61 ± 0.09	1.97 ± 0.04
PLA–PEG_1000_–chitosan + AgNPs (80:10:10 + 0.5 wt%)	0.63 ± 0.12	2.11 ± 0.04	0.129 ± 0.003	2.63 ± 0.39	1.93 ± 0.01

**Table 6:** List of test organisms for fungal resistance test.

Species	Strain Number
*Aspergillus brasiliensis* Varga et al., 2007	VKM F-1119
*Aspergillus terreus* Thom 1918	VKM F-1025
*Aspergillus sojae* Sakaguchi et K. Yamada ex Murakami 1971	VKM F-2096
*Chaetomium globosum* Kunze 1817	VKM F-109
*Paecilomyces variotii* Bainier 1907	VKM F-378
*Penicillium chrysogenum* Thom 1910	VKM F-245
*Penicillium aurantiogriseum* Dierckx 1901	VKM F-265
*Penicillium pinophilum* Thom 1910	VKM F-1115
*Trichoderma virens* (J.H. Mill et al., 1957) Arx 1987	VKM F-1117

**Table 7:** List of test organisms for antibacterial activity test.

Species	Strain Number	Type of Microorganism
*Bacillus subtilis* (Ehrenberg 1835) Cohn 1872	VKM B-501	Gram-positive obligate aerobic bacterium
*Escherichia coli* (Migula 1895) Castellani and Chalmers 1919	VKM B-3674	Gram-negative facultative anaerobic bacterium
*Micrococcus luteus* (Schroeter 1872) Cohn 1872	VKM Ac-2230	Gram-positive obligate aerobic bacterium
*Clostridium sporogenes* (Metchnikoff 1908) Bergey et al. 1923	VKM B-2623	Gram-positive obligate anaerobic bacterium
*Groenewaldozyma auringiensis* (Santa Maria 1978) Kurtzman 2016	VKM Y-2927	Facultative anaerobic yeast

## Data Availability

The original contributions presented in this study are included in the article. Further inquiries can be directed to the corresponding author.
